# An Integrated Instrumentation System for Velocity, Concentration and Mass Flow Rate Measurement of Solid Particles Based on Electrostatic and Capacitance Sensors

**DOI:** 10.3390/s151229843

**Published:** 2015-12-10

**Authors:** Jian Li, Ming Kong, Chuanlong Xu, Shimin Wang, Ying Fan

**Affiliations:** 1 Key Laboratory of Energy Thermal Conversion and Control of Ministry of Education, School of Energy and Environment, Southeast University, Nanjing 210096, China; lijian.5401@163.com (J.L.); smwang@seu.edu.cn (S.W.); 2 College of Metrology and Measurement Engineering, China Jiliang University, Hangzhou 310018, China; mkong@cjlu.edu.cn; 3 School of Energy and Power Engineering, Lanzhou University of Technology, Lanzhou 730050, China; fanying4321@163.com

**Keywords:** integrated instrumentation system, electrostatic sensor, capacitance sensor, particle velocity, concentration, solid mass flow rate

## Abstract

The online and continuous measurement of velocity, concentration and mass flow rate of pneumatically conveyed solid particles for the high-efficiency utilization of energy and raw materials has become increasingly significant. In this paper, an integrated instrumentation system for the velocity, concentration and mass flow rate measurement of dense phase pneumatically conveyed solid particles based on electrostatic and capacitance sensorsis developed. The electrostatic sensors are used for particle mean velocity measurement in combination with the cross-correlation technique, while the capacitance sensor with helical surface-plate electrodes, which has relatively homogeneous sensitivity distribution, is employed for the measurement of particle concentration and its capacitance is measured by an electrostatic-immune AC-based circuit. The solid mass flow rate can be further calculated from the measured velocity and concentration. The developed instrumentation system for velocity and concentration measurement is verified and calibrated on a pulley rig and through static experiments, respectively. Finally the system is evaluated with glass beads on a gravity-fed rig. The experimental results demonstrate that the system is capable of the accurate solid mass flow rate measurement, and the relative error is within −3%–8% for glass bead mass flow rates ranging from 0.13 kg/s to 0.9 kg/s.

## 1. Introduction

Pneumatic conveying is one of the most important ways for the transportation of pulverized fuel materials in many fields such as energy, chemical, metallurgy, pharmaceutical and foods industries. It is of great significance to achieve the accurate, online and continuous measurement of the velocity, concentration and mass flow rate of pneumatically conveyed solid particles for the high-efficiency utilization of energy and raw materials, operational safety and optimizedsystemcontrol.

A variety of advanced measurement techniques have been investigated and developed for the flow measurement of pneumatically conveyed solid particles based on different sensing principles [1], such as optical [2,3], digital imaging [4,5], acoustic [6,7], radiometric [8] and electric methods [9,10,11,12,13,14,15,16]. Among them, optical, digital imaging and acoustic techniques are capable of dilute phase particle flow measurement. However, to datefew of the proposed instrumentation systems can work in the harsh industrial environment, and these techniques do not function under dense phase conditions, where high solids loading is preferred to minimize the wear of the conveyor, solids undergo breakdown due to friction and collisions, and the transportationenergy consumption is high. The radiometric method is, in principle, an optional choice for the gas-solid flow measurement regardless of the particle concentration. However, it has a risk of radiation leakage and is usually costly due to the complicated sensor structure and radiation shielding required. Electric methods, mainly including electrostatic and capacitance sensing techniques, have been increasingly attractive due to their advantages of non-invasiveness, simple structure, low-cost, high reliability and non-radiation technology. Great advances have been made in particle velocity measurement in gas-solid flow by using electrostatic sensors along with signal processing algorithms such as cross-correlation [10,11,12,13,14] and spatial filtering [15,16,17] techniques, and encouraging results have been obtained in both laboratory and field experiments. On the other hand, the capacitance method has been successfully used for the phase concentration measurement in gas-liquid and gas-solid two-phase flows with optimized sensor structures [18,19] based on the variations of equivalent electricalpermittivitywith different phase concentrations. Electrostatic and capacitance sensors also can be combined with process tomography, *i.e.*, electrostatic tomography (EST) and electrical capacitance tomography (ECT), to realize particle flow parameters measurement and “flow visualization” [9,20,21]. 

In this paper, an integrated instrumentation system for the velocity, concentration and mass flow rate measurement of dense phase pneumatically conveyed solid particles based on electrostatic and capacitance sensors is developed. Two identical ring electrostatic sensors are used for the particle mean velocity measurement in combination with the cross-correlation technique, while acapacitance sensor with helical surface-plate electrodes, which has relatively homogeneous sensitivity distribution, is employed for the particle concentrationmeasurement and its capacitance is measured by an electrostatic-immune AC-based circuit. The mass flow rate of solid particles is further inferred from the obtained particle velocity and concentration. Experimental results are presented to evaluate the performance of the developed system.

## 2. Measurement Principle

The mass flow rate of solid particles over the cross-section of a pneumatic pipe can be inferred from the particle velocity and concentration:

(1) M=ρ⋅A⋅v⋅β

where *M* is the mass flow rate of solid particles, *ρ* is particle density, *A* is the cross-sectional area of the conveying pipe, *v* is the mean particle velocity and *β* is the mean volume concentration. If the particle velocity and concentration can be measured accurately, the solid mass flow rate could be calculated from Equation (1).

### 2.1. Particle Velocity Measurement Based on Electrostatic Sensors

Pneumatically conveyed solid particles are charged due to friction and collisions between the particles and the pipe wall, which is one of the inherent characteristics of gas-solid flows. Based on particle charging, various electrostatic sensors have been developed for particle flow parameter measurement [9,10,11,12,13,15,16,17], especially for the particle velocity measurement when combined with a cross-correlation signal processing algorithm. Two identical electrostatic sensors are essential for the cross-correlation velocity measurement and are usually arranged on the pipe with a certain axial spacing (*L*). Two corresponding electrostatic signals can be obtained by suitable electronic circuits connected to the sensors. When the particles flow moves along the axial direction of the pipe from the upstream sensor to the downstream sensor, the output signals from the two sensors are similar with each other, but have a time delay (the transit time). From the cross-correlation function of the two signals, the maximum value can be found and its time coordinate corresponds to the transit time (*τ*). As a consequence, particle velocity can be calculated by:

(2) v=L/τ

In practical application, the sampling frequency should be selected properly according to the frequency characteristic of the electrostatic sensor and the requirement of the relative error in the transit time measurement [22]. The signal bandwidth of the electrostatic signal is dependent on the particle velocity, the width of the sensor electrode and geometric size of the pipe. The sampling frequency should be more than twice the signal bandwidth according to the Nyquist–Shannon sampling theorem. The transit time is deduced according to the maximum valueof the correlation function and itsreal value maynot be located at the discrete points of the correlation function. The relative error in the transit time measurement is related to particle velocity, axial spacing of the two sensors and the sampling frequency. A lower relative error in the transit time measurement requires a higher sampling frequency, but this is not always necessary due to the inherent instability of particle flows. The tolerance of the relative error of the transit time should be set by taking the real application requirements into account. Another important parameter for the cross-correlation calculation is the integration time. It should be more than 10 times the transit time [23]. Although a longer integration time is useful for the accurate measurement of the transit time, velocity measurement is time-consuming and the response of the system becomes slow.

### 2.2. Particle Concentration Measurement with Capacitance Sensor

The equivalent electrical permittivity of a two-phase mixture varies with different constituent contents. When a gas-solid two-phase flow passes through the sensing space of a capacitance sensor, the capacitance between the metal electrodes of the sensor will change with the solid volume concentration. As a consequence, the particle concentration can be derived from thecapacitance change of the sensor.

For the capacitance sensor applied to gas-solid flow measurement, its capacitance (*C_e_*) is determined by the geometric size and the equivalent relative permittivity (*ε_e_*) of the media within its sensing space [24]:

(3) $$C_{e}=C_{e0}f(\varepsilon_{e})$$

where *C_e_*_0_ is the capacitance of the sensor when the pipe is full of gas, and *f*(*ε_e_*) is a function. Usually, the relative permittivity of gas can be approximately regarded as 1, and thus *C_e0_* is completely determined by the geometric size of the sensor. However, the expression of *f*(*ε_e_*) is related to the sensor structure, and is not a proportional function except for the parallel-capacitance model [24]. On the other hand, it is also challenging to calculate the equivalent relative permittivity of the gas-solid mixture, which depends on the relative permittivity of gas and solid, and the particle concentration distribution (*β_V_*(*x,y,z*)):

(4) $$\varepsilon_{e}=g(\varepsilon_{g},\varepsilon_{s},\beta_{V}(x,y,z))$$

where *ε_g_* and *ε_s_* are the relative permittivities of gas and solid particles, respectively, and *g*(*ε_g_*, *ε_s_*, *β_V_*(*x,y,z*)) is a function. So *C_e_* can be written as:

(5) $$C_{e}=C_{e0}h(\varepsilon_{g},\varepsilon_{s},\beta_{V}(x,y,z))$$

where *h*(*ε_g_*, *ε_s_*, *β_V_*(*x,y,z*)) is a function. At present, it is difficult to achieve the analytical solution of Equation (5). In capacitance sensor applications, its structure and the type of the media are known, so the equivalent capacitance is mainly dependent on the particle concentration and its distribution (flow pattern) over the cross-section of the pipe. This is because the capacitance sensor has aninhomogeneous sensitivity distribution in its sensing space, which may lead to measurement errorsin particle concentration measurement despitethe similar concentration.

To reduce the measurement error, the sensitivity of a capacitance sensor must beperfectly homogeneous. Many trials have been carried out to optimize and develop a suitable sensor structure to ensure ahomogeneous sensitivity distribution [18,25,26,27]. The results prove that the capacitance sensor with helical surface-plate electrodes has a more homogeneous sensitivity, which makes it immune to flow patterns. After calibration, the capacitance sensor with helical surface-plate electrodes can be used for the accurate concentration measurement of solid particles.

## 3. Design of the Integrated Instrumentation System

**Figure 1:**
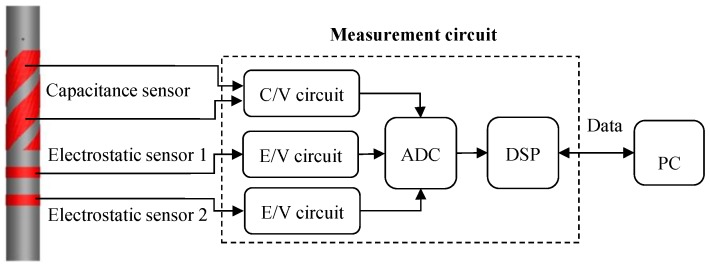
Integrated instrumentation system based on electrostatic and capacitance sensors.

Figure 1 shows a schematic diagram of the proposed integrated instrumentation system. The system is comprised of two ring-shaped electrostatic sensors for particle velocity measurement, one capacitance sensor with helical surface-plate electrodes for particle concentration measurement, one measurement circuit based on a digital signal processor (DSP) and a personal computer (PC).

**Figure 2:**
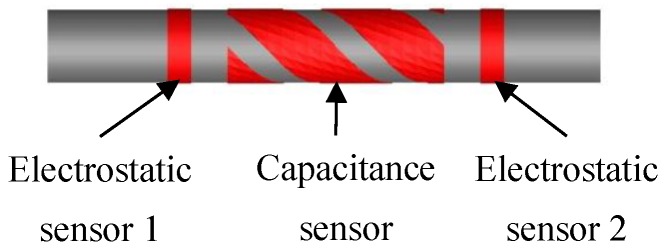
Sensor configuration.

The electrodes of the electrostatic and capacitance sensors are made of copper with a thickness of 0.2 mm, and they are mounted on the outer surface of a plexiglass pipe. All the electrodes are covered in a metal shield to isolate them from any external electromagnetic interference. The inner and outer diameters of the plexiglass pipe are 50 mm and 60 mm, respectively. The axial width of the electrodes of the electrostatic sensors is 20 mm and the axial spacing between the electrodes is 40 mm. The helical surface-plate electrodes of the capacitance sensor have a axial width of 55 mm and twist one turn along the pipe surface with a pitch of π × 60 mm. In Figure 1, the two electrostatic sensors are arranged on the same side of the capacitance sensor. If the integrated system is applied to the particle measurement in a vertical pipe, the correlation velocity from the electrostatic sensors obviously deviates from the mean velocity of particles within the capacitance sensor due to acceleration effect. This will lead to a measurement error in the solid mass flow rate. Another option is to configure the two electrostatic sensors on each side of the capacitance sensor, as shown in Figure 2. In this case, the correlation velocity accurately represents the mean velocity of the solid particles within the sensing space of the capacitance sensor. However, the axial spacing of the two electrostatic sensors then increases to 250 mm, much longer than that in Figure 1. A large axial spacing may decrease the reliability of the velocity measurement, especially under the unstable flow conditions seen at low velocity. Therefore the practical flow conditions should be considered for the configuration of the sensors in the integrated instrumentation system.

**Figure 3:**
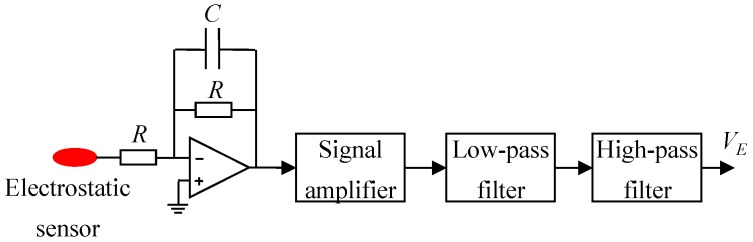
Charge to voltage conversion (E/V) circuit.

**Figure 4:**
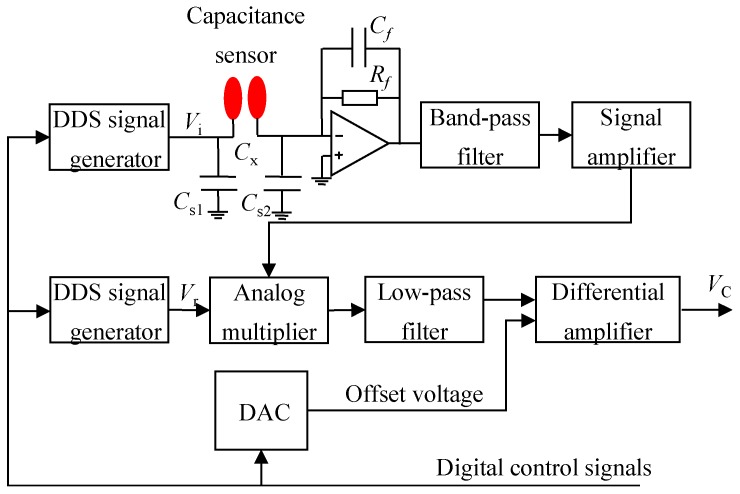
Capacitance to voltage conversion (C/V) circuit.

The DSP-based measurement circuit mainly consists of two electrostatic charge to voltage conversion (E/V) circuits, one capacitance to voltage conversion (C/V) circuit, one multi-channel analog to digital converter (ADC), and a DSP. The E/V circuits (Figure 3) are connected to the electrostatic sensors for the electrostatic signal conditioning, while the electrostatic-immune C/V circuit (Figure 4) based on an AC-based method is used for the capacitance measurement [28,29]. The electrostatic and capacitance signals are sampled by the DSP via the ADC. Particle velocity, concentration and mass flow rate are calculated in the DSP, and then sent to PC by a serial port for storage and display. In the C/V circuit, a band-pass filter is used to eliminate the influence of particle charging on the capacitance measurement, and a differential amplifier is to balance the standing capacitance of the capacitance measurement system, only leaving the capacitance change to be further amplified and processed.

**Figure 5:**
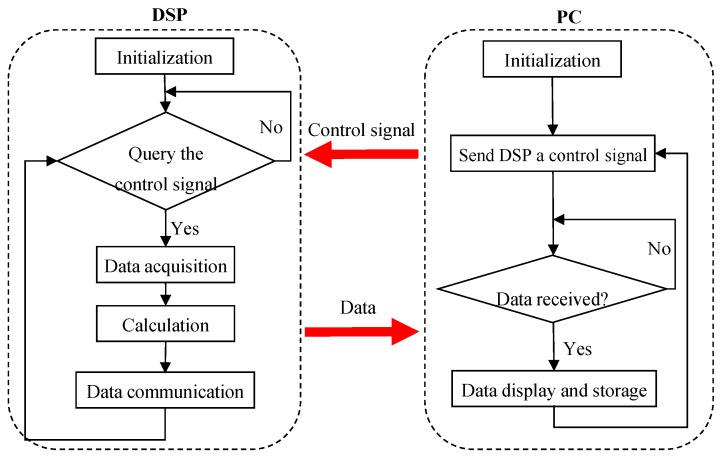
Flow chart of the software of the measurement system.

The flow chartof the software of the measurement system is shown in Figure 5. After the initialization of the DSP and PC, the PC sends a control signal to DSP. If the signal is accepted, the DSP will sample the electrostatic and capacitance signals at the preset sampling frequency and integration time, and then the flow parameters including particle velocity, concentration and mass flow rate are calculated. Subsequently, the DSP sends the flow parameters to the PC and waits for the next control signal. Once the PC receives the parameters, it will send the next control signal to the DSP immediately after completing the display and storage of the parameters. The number of data sampling points in the experiments is 2000, and the corresponding response time of the measurement system is about 1.5 s.

## 4. Experimental Results and Discussion

### 4.1. Tests on a Pulley Rig

**Figure 6:**
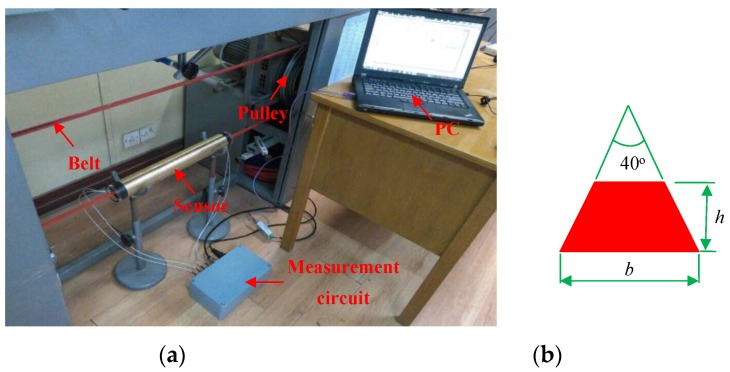
V-belt pulley rig: (**a**) Photograph; (**b**) Cross-section of the belt.

The instrumentation system for velocity andconcentration measurement is first tested on a V-belt pulley rig, as demonstrated in Figure 6. The belt is made of polyurethane and has a trapezoidal cross-section. Three types of belt with different sizes (Table 1) are used in the experiments. The diameter of the pulley (*D*) can be selected as 320 mm or 255 mm and the rotation speed can be adjusted by a variable-frequency driving motor. During the operation of the rig, the charge will be generated on the belt. Even though the motion of the belt is apparently different from the particle flow, the electrostatic induction phenomena, caused by the charge carried by belt and particles, respectively, are the same in essence. Another advantage of the rig is that the sectional area of the belt is fixed during the system operation, *i.e.*, the solid concentration does not change with the velocity, which is beneficial to assess the performance of the C/V circuit for the concentration measurement.

**Table 1:** Belt types.

Type	*b*	*h*	Area	Volume Fraction
#1	8 mm	5 mm	43.9 mm^2^	2.23%
#2	13 mm	8 mm	80.7 mm^2^	4.11%
#3	17 mm	11 mm	143.0 mm^2^	7.28%

The rotation speed of the pulley (*r*) is measured by a frequency counter, and thus the velocity of the belt (*v_B_*) can be calculated. It is regarded as the reference velocity:

(6) $$v_{B}=\pi \cdot D\cdot r$$

The range of the rotation speed of the pulley is about 400 r/min–1300 r/min, which corresponds to a belt velocity range of 5.3 m/s–21.8 m/s. The axial spacing of the two electrostatic sensors is 40 mm. To assure that the relative measurement error in the transit time is less than ±2%, the sampling frequency is set at 20 kHz. The integration time is 0.1 s.

**Figure 7:**
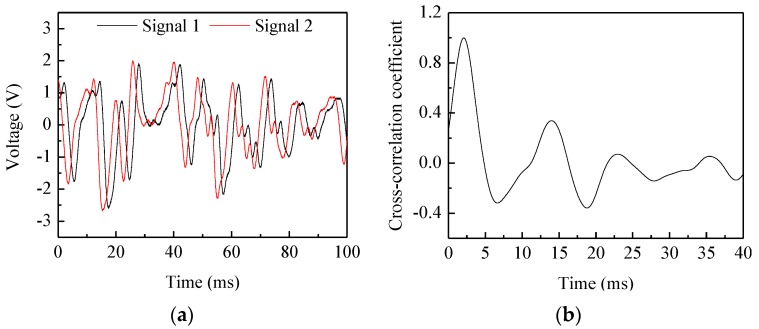
Typical electrostatic signals and their correlation function: (**a**) Electrostatic signals; (**b**) Correlation function.

**Figure 8:**
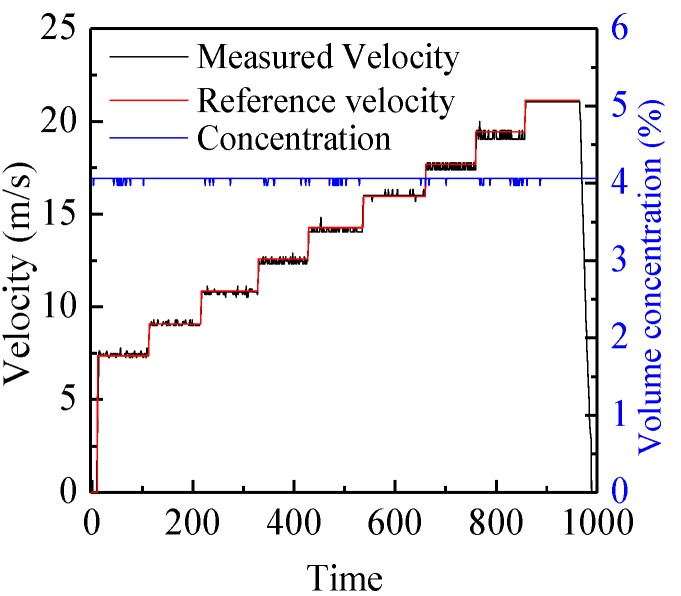
Continuous measurement results of velocity and concentration.

Figure 7 shows the typical electrostatic signals and their correlation function for belt #2 in the center of the pipe when the rotation speed of the pulley is 1164 r/min and its diameter is 320 mm. It can be seen that the two signals have a high similarity except for a time delay. An obvious peak can be observed in the correlation function and its time coordinate (2.08 ms) corresponds to the transit time, so the correlation velocity is 19.23 m/s, which is consistent with the reference velocity of 19.5 m/s. Figure 8 demonstrates the continuous measurement results of velocity and concentration for belt #2 in the center of the pipe at different rotation speeds of the pulley. The increasing rotation speed leads to a larger reference velocity, and thus larger measured velocity. The measured velocity and reference velocity are highly in agreement, even though there is a very small fluctuation due to the shaking and stretching of the belt. It also can be seen that the measured concentration is almost fixed as the ratio of the sectional area of the belt to that of the pipe is a constant for different belt velocities. Similar measurement results for the three types of belt at different rotation speeds of pulley are also obtained, which indicates that the C/V circuit has a good stability for particle concentration measurement regardless of particle velocities.

**Figure 9:**
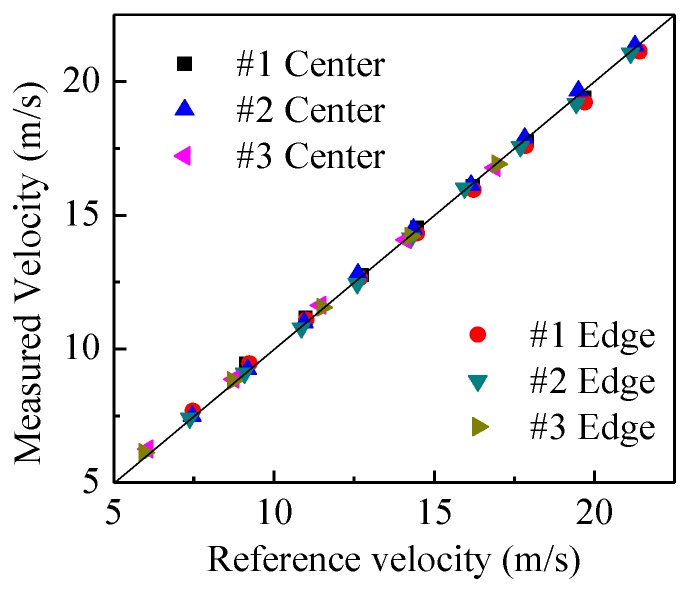
Comparison of measured and reference velocities.

**Figure 10:**
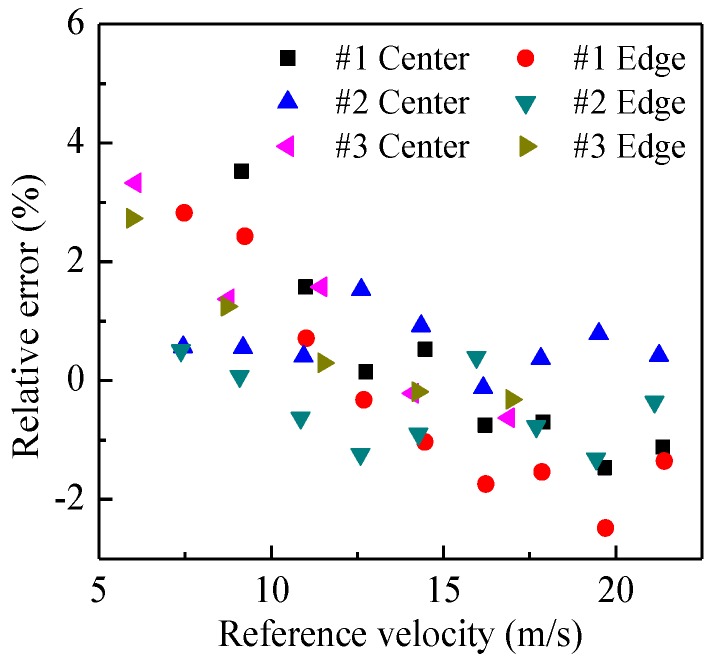
Relative measurement error of velocity.

**Figure 11:**
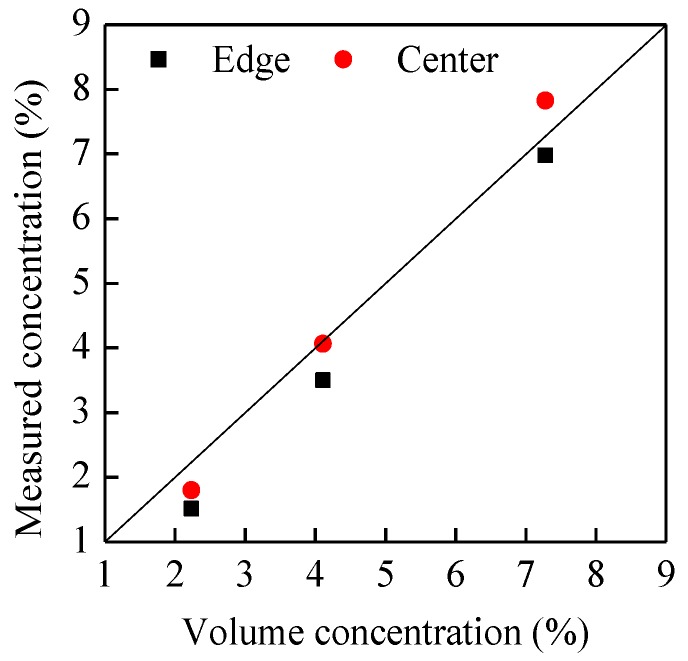
Measured volume concentration.

Figure 9 shows the comparisons of the measured velocities and reference velocities for the three types of belt at different rotation speeds of the pulley. It is obvious that the measured velocities are very close to the reference velocities, no matter whether the belt is in the center of the pipe or near the pipe wall (edge location). The relative error is within the range of −2%–4%, as shown in Figure 10. It verifies that the developed system can be used for accurate particle velocity measurement. Figure 11 demonstrates the measured volume concentrations for the three types of the belt. It can be seen that the measured concentrations for the location of “center” are always larger than those for the location of “edge”, and the maximum relative error of the measured concentrations to the true value is about 32.1%. This is because the geometric size of the capacitance sensor is optimized based on glass, which will be introduced in Section 4.2, and the polyurethane has a different relative permittivity fromglass, so the calibrated results based on glass cannot be used directly for other materials. The results also indicate that the optimal geometric size of capacitance sensor changes and recalibration of the concentration measurement system must be conducted for different materials.

### 4.2. Calibration of the Capacitance Sensor

**Figure 12:**
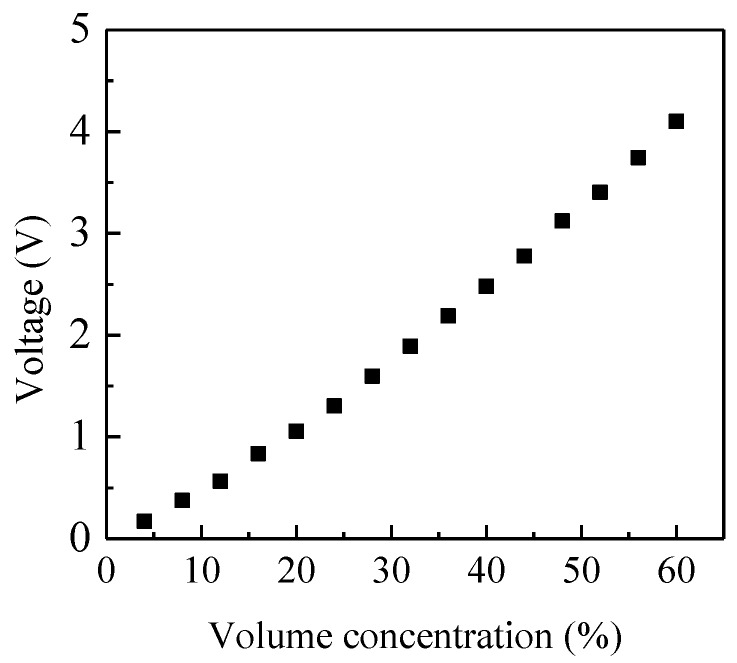
Average output voltage of the C/V circuit *vs*. concentration.

**Figure 13:**
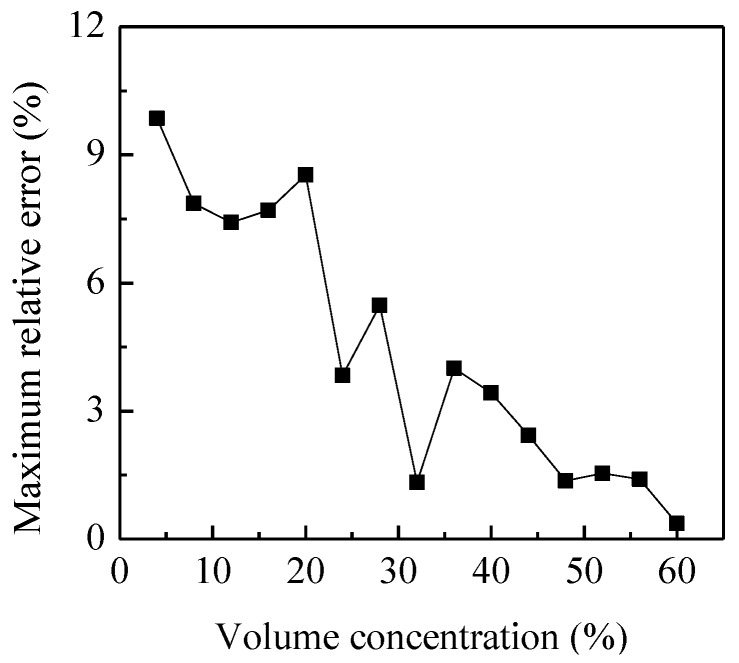
Maximum relative errorof the output voltageto the average value.

A capacitance sensor with helical electrodes has the same sensitivity on the circumference with a given radius [22]. The main factors affecting the sensitivity distribution are the pitch and axial width of the sensor electrodes. Usually, the pitch should be selected to be long enough in order to neglect the fringe effect. Then a homogeneous sensitivity distribution of the sensor can be obtained by optimizing the axial width of the electrode. In this paper, the pitch of the electrode is selected as π × 60 mm, and the axial width is optimized by trial and error. A sequence of axial widths are tested by putting a 10 mm glass bar (parallel to the axial direction of the pipe) in the integrated system to compare the sensitivities on different locations of the pipe cross-section. As a result, the optimal axial width is 55 mm for glass. It should be noticed that the optimal axial width of the capacitance sensor varies with materials due to their different relative permittivities.When the different numbers of 10 mm glass bars are put into the pipe, the volume concentration of solid changes and its relationship with the output voltage of the C/V circuit can be calibrated. Figure 12 shows the output voltage of the C/V circuit for different volume concentration of glass. They are the average values of the output voltages when glass bars are parallel to the axis of the pipe in different optional positions over the cross-section. Figure 13 illustrates the maximum relative errors of the output voltage to the average value when glass bars are put into the pipe randomly.

It can be seen that the output voltage of the measurement circuit increases with the volume concentration, but the relationship is not linear. Moreover, the maximum relative error is within ±10% anddecreases with the increasing volume concentration. By curve fitting, the relationship between the output voltage of the C/V circuit (*V_C_*) and the volume concentration of glass can be expressed by a second-order polynomial:

(7) $$\beta =-0.01V_{C}^{2}+0.194V_{C}$$

Therefore, the volume concentration can be easily calculated from the output voltage of the C/V circuit according to Equation (7).

### 4.3. Measurements on a Gravity-Fed Rig

**Figure 14:**
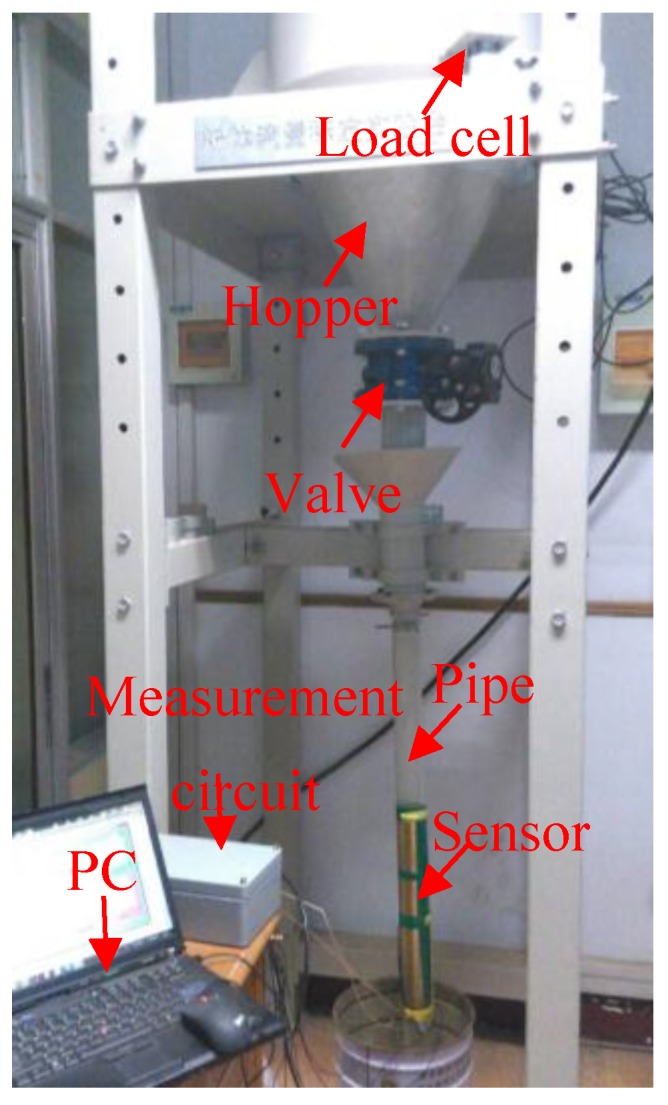
Gravity-fed rig.

Experiments are also carried out on a gravity-fed rig (Figure 14) to evaluate the effectiveness of the developed instrumentation system. The particles are glass beads with an average diameter of 3 mm, and thus Equation (7) can be directly used for the concentration measurement. 

**Figure 15:**
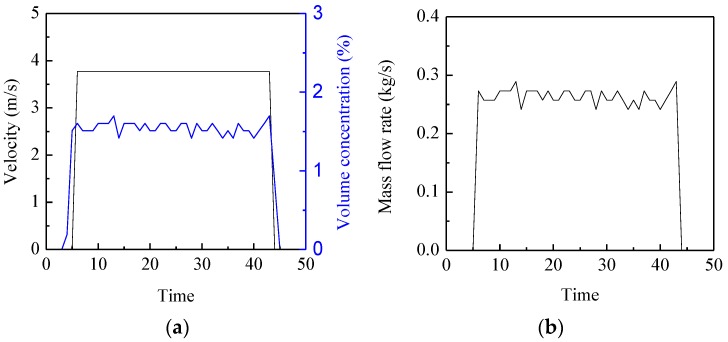
Continuous measurement results for a typical valve opening: (**a**) Particle velocity and concentration; (**b**) Mass flow rate.

**Figure 16:**
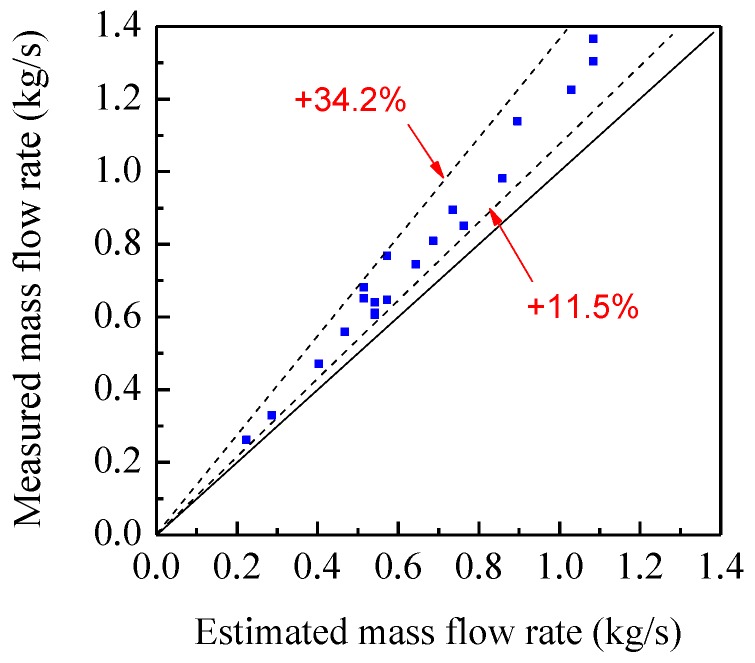
Comparison of the measured mass flow ratewith the estimated value.

In experiments, the mass flow rate of glass beads is adjusted by a valve at the outlet of the hopper. A load cell is installed to record the variation of the mass of the glass beads in the hopper. The average mass flow rate is calculated from its output signal and regarded as the reference value [15]. Due to the limitation of the distance between the sensors and the hopper, the particle velocity is less than 5 m/s. As for the configuration of the electrostatic and capacitance sensors in Figure 1, the sampling frequency and integration time are selected as 5 kHz and 0.4 s, respectively. Figure 15 shows the continuous measurement results of the particle velocity, concentration and mass flow rate for a typical valve opening. It can be seen that both the measured velocity and concentration have good stability, and hence the mass flow rate. Figure 16 shows the comparison between the measured mass flow rate and the estimated value from the load cells. It can be seen that the measured value is obviously larger than the estimated value. This is because the two electrostatic sensors are configured beneath the capacitance sensor. The acceleration of the particles passing through the sensors due to gravity could not be neglected. Therefore, the measured velocity is more than the mean velocity when particles flow through the capacitance sensor, which results in a larger value of the measured mass flow rate.

To reduce the impact of the accelerated motion of particles, the two electrostatic sensors should be installed to each side of the capacitance sensor, as shown in Figure 2. As the axial spacing of the two electrostatic sensors is 250 mm, the sampling frequency and integration time are reselected as 1 kHz and 1 s, respectively. 

**Figure 17:**
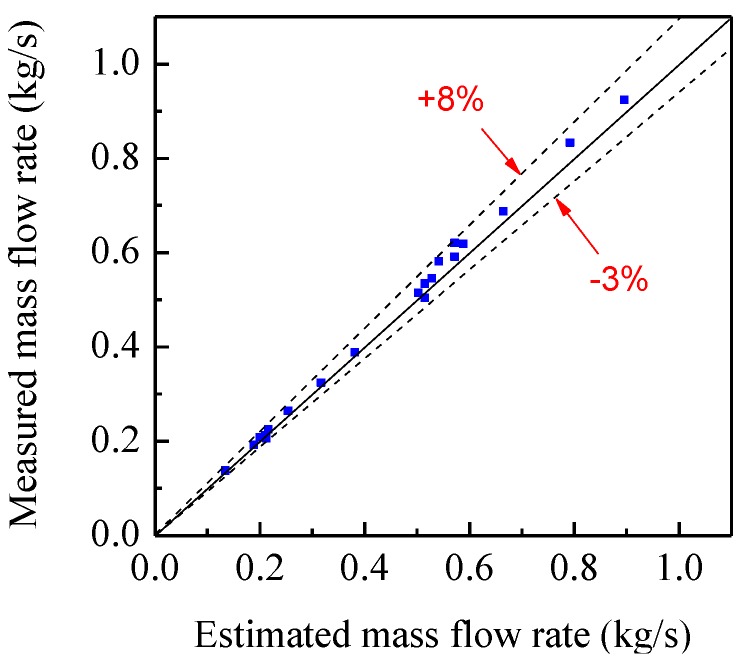
Comparison of the measured mass flow ratewith the estimated value for the sensor configuration in Figure 2.

Figure 17 shows the comparison of the average measured mass flow rate with the estimated value. It is can be seen that they have a good consistence. The relative error is −3%–8%, much better than that in Figure 16. As the particle concentration is withinabout 1%–8% in experiments, the measurement results are acceptable due to the relatively higher measurement error for low concentration as calibrated in Figure 13. It is expected that more accurate results can be obtained for higher concentrations of solids particles.

## 5. Conclusions

An integrated instrumentation system based on electrostatic andcapacitance sensors has been developed in this paper. Experiments have been carried out on a V-belt pulley rig and a gravity-fed rig, respectively. The experimental results demonstrated that the measurement error of the solid mass flow rate is −3%–8% within the range of 0.13 kg/s–0.9 kg/s for glass beads. It proves that the system is capable of the online and continuous measurement of particle velocity, concentration and mass flow rate. Although the range of particle volume concentrations in the experiments is within about 1%–8%, it is expected that more accurate results can be obtained for higher concentrations of solids particles. Moreover, the instrumentation system is designed based on DSP and can be directly applied in industrial environments to provide particle flow parameters to system control units. In future work we will evaluate the performance of the instrumentation system on a dense phase pneumatic conveyor of pulverized fuel particles.
